# Suco de Beterraba Pode Ser um Ingrediente Dietético para Proteger o Endotélio Vascular

**DOI:** 10.36660/abc.20220906

**Published:** 2023-01-31

**Authors:** Roberto J. S. Franco

**Affiliations:** 1 Faculdade de Medicina de Botucatu Universidade Estadual Paulista São Paulo SP Brasil Faculdade de Medicina de Botucatu - Universidade Estadual Paulista – UNESP, São Paulo, SP – Brasil

**Keywords:** Beta Vulgaris, Endotélio Vascular, Reatividade, Hipertensão, Óxido Nítrico

As primeiras Recomendações Dietéticas originaram-se em 1941 por ordem do Presidente Franklin Roosevelt, quando convocou a “Conferência Nacional de Nutrição em Defesa” para garantir uma população apta para a guerra, minimizando as doenças por deficiência de nutrientes.^1^ No mesmo ano, a Associação Médica Americana declarou que “a pesquisa em nutrição deve ser encorajada” com objetivos primários de “estimar as quantidades de nutrientes essenciais nos alimentos”, “deteção de estados de deficiência nutricional”, e determinação mais precisa de “ótimos e mínimos requisitos” para cada nutriente.^2^

Em humanos, após absorção através da parede do estômago, 25% do nitrato consumido entra na circulação entero-salivar onde é reduzido a nitrito (NO_2_) por nitrato redutases bacterianas de anaeróbios facultativos na superfície dorsal da língua.^3 , 4^ Este nitrito é engolido e no ambiente ácido do estômago é reduzido a óxido nítrico (NO) ou entra novamente na circulação como nitrito. Desse modo, o nitrato dietético pode representar uma fonte intravascular da molécula vasoprotetora pleiotrópica NO e atua como potente dilatador, sobre a pressão arterial e retarda a aterogênese inibindo o recrutamento de células inflamatórias e a agregação plaquetária. Assim, protegendo o endotélio vascular, influencia numerosas patologias cardiovasculares (pré-hipertensão, hipertensão, aterosclerose, e acidente vascular cerebral) associadas com disfunção endotelial e diminuição da bioatividade do NO.^5^ Recentemente, estudos demonstraram que o nitrito confere proteção marcada contra lesão de isquemia/reperfusão (I/R) no miocárdio, rim, pulmão, e vasculatura cerebral.^5 , 6^

Nesse número dos Arquivos Brasileiros de Cardiologia foi publicado um estudo^7^ para avaliar os efeitos agudos da ingestão dietética de NO_3_- contido em 500 ml de suco de beterraba (SB) sobre a pressão arterial e a função endotelial em pacientes hipertensos tratados. Foram incluídos pacientes hipertensos em uso regular de antihipertensivos admitidos para estudo randomizado, cruzado, placebo controlado. Após avaliação e randomização os indivíduos receberam SB ou água como controle (C) e permaneceram em repouso por 150 min., tempo de pico de ação do NO_3_- e NO_2_- na circulação sanguínea e depois foram reavaliados. Além da coleta de dados bioquímicos, antropométricos, pressão arterial e risco cardiovascular, foi feita a reatividade microvascular com equipamento específico combinado com hiperemia reativa pós-oclusiva para redução contínua de alterações de perfusão cutânea dependentes do endotélio microvascular. Para variáveis hemodinâmicas centrais foram analisados de forma não invasiva e com aparelho de tonometria a pressão sistólica aórtica central, o pulso aórtico, o aumento, o índice de aumento e a duração de ejeção (DE). Esse equipamento permitiu calcular o índice de variabilidade subendocárdica (RVSP) que fornece estimativa da perfusão miocárdica em relação ao trabalho cardíaco e é preditivo da reserva de fluxo coronariano. Quanto menor a RVSP, menor a perfusão cardíaca que se relaciona à rigidez arterial, fator de envelhecimento vascular.

O grupo SC teve aumento significante nos níveis séricos de NO_3_- e NO_2_-, três vezes maior que os valores basais.

Houve aumento significativo na taxa de viabilidade subendocárdica (RVSE; 149±25 vs. 165±30%, p<0,001) e redução na duração da ejeção (DE; 37±4 vs. 34±4%, p<0,001) na fase beterraba, mas nenhuma diferença significativa de RVSE na fase controle. A % de aumento na perfusão (155 vs. 159%, p=0,042) cresceu significativamente na fase beterraba, o que não foi observado na fase controle.

Como conclusão a ingestão de SB resultou em benefícios agudos nos parâmetros vasculares em indivíduos hipertensos, levando a uma maior viabilidade subendocárdica e desempenho na contração miocárdica além da melhora da função endotelial. Este foi o primeiro estudo que aplicou métodos diferentes para avaliar parâmetros vasculares e demonstrar efeitos benéficos da ingestão única de SB em adultos hipertensos tratados. Cabe aos autores comprovarem esses benefícios do suco de beterraba administrados por um período de médio a longo.
