# Supplementary material for: Suco de Beterraba Pode Ser um Ingrediente Dietético para Proteger o Endotélio Vascular
Source: Arq Bras Cardiol. 2023 Jan 31;120(1):e20220906. [Article in Portuguese] doi: 10.36660/abc.20220906 (PMC10389108; doi:10.36660/abc.20220906)
Supplement: Translation

# Translation: Beetroot Juice May Be a Dietary Ingredient to Protect Vascular Endothelium

**Authors:** Roberto J. S. Franco

**Affiliations:** 1 Brasil Faculdade de Medicina de Botucatu - Universidade Estadual Paulista – UNESP, São Paulo, SP – Brasil

**Keywords:** Beta Vulgaris, Endothelium Vascular, Reactivity, Stability, Oxide Nitric

The first Dietary Recommendations originated in 1941 by order of President Franklin Roosevelt, when he convened the “National Conference on Nutrition in Defense” to guarantee a population fit for war, minimizing diseases due to deficiency of nutrients.^1^In the same year, the American Medical Association declared that “nutrition research should be encouraged” with primary objectives of “estimating the amounts of essential nutrients in foods,” “detection of nutritional deficiency states,” and more precise determination of “optimal and minimum requirements” for each nutrient.^2^

In humans, after absorption through the stomach wall, 25% of the nitrate consumed enters the entero-salivary circulation, where it is reduced to nitrite (NO_2_) by bacterial nitrate reductases of facultative anaerobes on the dorsal surface of the tongue.^3 , 4^This nitrite is swallowed and, in the stomach’s acidic environment, is reduced to nitric oxide (NO) or re-enters the circulation as nitrite. Thus, dietary nitrate may represent an intravascular source of the pleiotropic vasoprotective molecule NO and acts as a potent dilator of blood pressure and delays atherogenesis by inhibiting the recruitment of inflammatory cells and platelet aggregation. Thus, protecting the vascular endothelium influences numerous cardiovascular pathologies (prehypertension, hypertension, atherosclerosis, and stroke) associated with endothelial dysfunction and decreased NO^5^bioactivity. Recently, studies have shown that nitrite confers marked protection against ischemia/reperfusion (I/R) injury to the myocardium, kidney, lung, and cerebral vasculature.^5 , 6^

In this issue of the *Arquivos Brasileiros de Cardiologia* , a study^7^was published to evaluate the acute effects of dietary intake of NO_3_‾ contained in 500 ml of beetroot juice (BJ) on blood pressure and endothelial function in treated hypertensive patients. Hypertensive patients regularly using antihypertensives admitted to a randomized, crossover, placebo-controlled study were included. After evaluation and randomization, the individuals received BJ or water as a control (C) and remained at rest for 150 min., time of peak action of NO_3_- and NO_2_- in the bloodstream, and then were reassessed. In addition to collecting biochemical, anthropometric, blood pressure, and cardiovascular risk data, microvascular reactivity was performed with specific equipment combined with post-occlusive reactive hyperemia for continuous reduction of changes in cutaneous perfusion dependent on the microvascular endothelium. For central hemodynamic variables, central aortic systolic pressure, aortic pulse, augmentation, augmentation index, and ejection duration (ED) were analyzed non-invasively and using a tonometry device. This equipment made it possible to calculate the subendocardial variability index (SEVR), which provides an estimate of myocardial perfusion concerning cardiac work and is predictive of coronary flow reserve. The lower the SEVR, the lower the cardiac perfusion, which is related to arterial stiffness, a vascular aging factor.

The SC group significantly increased serum levels of NO_3_- and NO_2_-, three times greater than baseline values.

There was a significant increase in the subendocardial viability rate (SEVR; 149±25 vs. 165±30%, p<0.001) and a reduction in ejection duration (ED; 37±4 vs. 34±4%, p<0.001) in the beetroot, but no significant SEVR difference in the control phase. The % increase in perfusion (155 vs. 159%, p=0.042) increased significantly in the beet phase, which was not observed in the control phase.

In conclusion, SB ingestion resulted in acute benefits in vascular parameters in hypertensive individuals, leading to greater subendocardial viability and performance in myocardial contraction and improved endothelial function. This was the first study that applied different methods to evaluate vascular parameters and demonstrate the beneficial effects of a single BJ intake in treated hypertensive adults. It is up to the authors to prove these benefits of beet juice administered for a medium to long period.
